# Sulcal artery syndrome; a pain predominant presentation

**DOI:** 10.1093/bjrcr/uaaf062

**Published:** 2025-12-08

**Authors:** Liam Robert Smith, Lay Kun Kho, Ferry Dharsono, David Prentice

**Affiliations:** Royal Perth Hospital, East Metropolitan Health Service, Perth, Western Australia, 6000, Australia; Royal Perth Hospital, East Metropolitan Health Service, Perth, Western Australia, 6000, Australia; Neurological Intervention & Imaging Service of Western Australia, Perth, Western Australia, 6000, Australia; Retired

**Keywords:** spinal cord infarction, anterior spinal artery, posterior spinal artery, radicular artery, vertebral artery, spinal cord sulcal syndrome

## Abstract

A 41-year-old man with a neonatal history of Erb’s palsy presented with severe bilateral gnawing radicular arm pain. A detailed neurological examination detected no abnormality. Imaging revealed right hemi spinal cord T2 hyperintensity together with an occluded left vertebral artery to the V4 segment. The clinical syndromes and mechanisms of spinal cord infarction are discussed. A hypothesis for the lack of sensorimotor signs is put forward.

## Introduction

Spinal cord infarction (SCI) is the rarest cause of stroke, accounting for 1% of strokes.^1^^,^^2^ Clinically, it presents with an abrupt onset of pain at the level of the spinal cord involved, followed by motor and sensory symptoms usually within 24 hours. Often in the cervical cord, there is a trigger with sudden movement of the neck, trauma or abnormal position. The commonest causes of spinal cord infarction (SCI) are radicular artery disease from proximal atherosclerosis or dissection, syphilis, fibrocartilaginous emboli, cardiac emboli, and vasculitis (giant cell and Takayasu’s).^3–5^ The recognized syndromes are (1) anterior SCI with bilateral arm weakness alone or quadriparesis together with a spinothalamic deficit to the spinal level of the stroke; (2) posterior cord infarction with bilateral, or unilateral position sensory loss to the spinal level and occasionally some mild motor weakness; (3) sulcal artery or hemi-cord syndrome with unilateral hemiplegia and contralateral spinothalamic loss; (4) transverse spinal cord syndrome with loss of all modalities below the spinal level which typically involves the thoracic or lumbar spinal cord; (5) central cord syndrome with pain and temperature sensory loss.^5^ The blood supply to the spinal cord is complex, with many variants and will be detailed in the discussion.^6^

Vertebral artery dissection (VAD) is a common cause of stroke in young- or middle-aged patients. VAD commonly causes neck pain and stroke in the posterior fossa with medullary and cerebellar involvement.^7^ SCI can occur in up to 10% of VAD, and contributes to 4%-10% of SCI.^8^^,^^9^ Bilateral chronic vertebral artery occlusion is not uncommon in the elderly, but spinal cord infarction is rare, as there is an extensive collateral blood supply to the cervical cord.

## Case summary

A 41-year-old male with a background of Type One Diabetes Mellitus and a left Erb’s Palsy secondary to neonatal brachial plexus injury presented with severe gnawing/aching pain in his neck, left shoulder and right arm. Pain severity was 9/10 and had no associated alleviating or exacerbating factors. He had no preceding trauma, illness, or new/excess physical activity or strain.

Upon examination, he was tachypnoeic at 20 breaths/min and hypertensive (148/94 mmHg). His ECG and troponins were normal. The rest of his examination, aside from his Erb’s palsy, was normal. The pain settled two hours after paracetamol, and he was discharged.

He re-presented to the Emergency Department after awaking at 0100 with a repeat episode of a similar severe pain in both arms, with a right predominance. He also experienced diaphoresis and left-sided aching chest pain of 6/10 severity. Between presentations, he had remained inactive, with intermittent mild aching discomfort in his arms that settled with simple analgesia. Once again, he had no other symptoms on systems review.

Examination revealed tachypnoea (20 breaths/min) and hypertension (165/90 mmHg). Again, the general examination was normal. Blood tests, including FBC, UEC, LFT, lipase, d-dimer and serial troponins were normal. Blood glucose was 12.5 mmol/L. Chest X-ray was unremarkable. A CT-Thoracic Aortogram to exclude aortic dissection was negative. His pain settled with analgesia over a period of hours, and he was discharged home.

During both episodes, there is no documentation of a formal neurological examination.

The patient then presented for the third time in 9 days, having been referred by his General Practitioner with a suspected vertebral artery dissection. He had episodic aching bilateral upper limb pain since discharge from the Emergency Department. He was referred by his GP for an MRI of his cervical spine, which demonstrated a loss of flow void of the left cervical and intradural vertebral artery in keeping with occlusion, with associated T2 central cord FLAIR hyperintensity between the levels of C5-C6, mainly on the right.

He was admitted to the Neurology Department. His only new symptom was intermittent numbness of the medial aspect of his right hand. A further episode of severe pain resolved with rest and paracetamol. The pain was described as extending from his neck and shoulders down the medial aspect of his right arm to the hand, and from the shoulder and neck down the volar aspect of the left arm. He described some intermittent anaesthesia of his right hand. Similarly, the pain had the same aching and gnawing character.

On cranial nerve examination, the only abnormal findings were a mild anisocoria with the right pupil 3 mm, and the left pupil 2.5 mm, and a partial left-sided ptosis consistent with a Horner’s syndrome attributed to his Erb’s palsy.

On examination, the right upper limb had normal tone and power. The left upper limb had reduced tone and power in the C5/6 motor distribution consistent with a known Erb’s palsy. His sensation was preserved and symmetric to sharp, soft, and thermal stimulation, as was his proprioception to bilateral distal interphalangeal joints, and without a sensory level. His reflexes were absent in the left upper limb, which was attributed to his brachial plexus injury, 1+ in the right brachioradialis and biceps, and augmentation was required to elicit the triceps reflex. He had no dysmetria or dysdiadochokinesis.

His lower limb neurological examination was unremarkable with symmetrical tone, power, and preserved sensation to sharp, soft, and thermal stimulation. Reflexes were symmetrically normal with downgoing planter reflexes, and preserved coordination. His gait was normal.

He was admitted and loaded with aspirin for possible vertebral artery dissection with SCI.

Thrombophilia, vasculitis, and infective screens were negative.

His CT-Brain with head and neck angiogram demonstrated complete occlusion of his left vertebral artery (Figure 2A), with a similar appearance of the vertebral artery origin noted when the CT-Thoracic Aortogram was re-reviewed.

**Figure 1.**
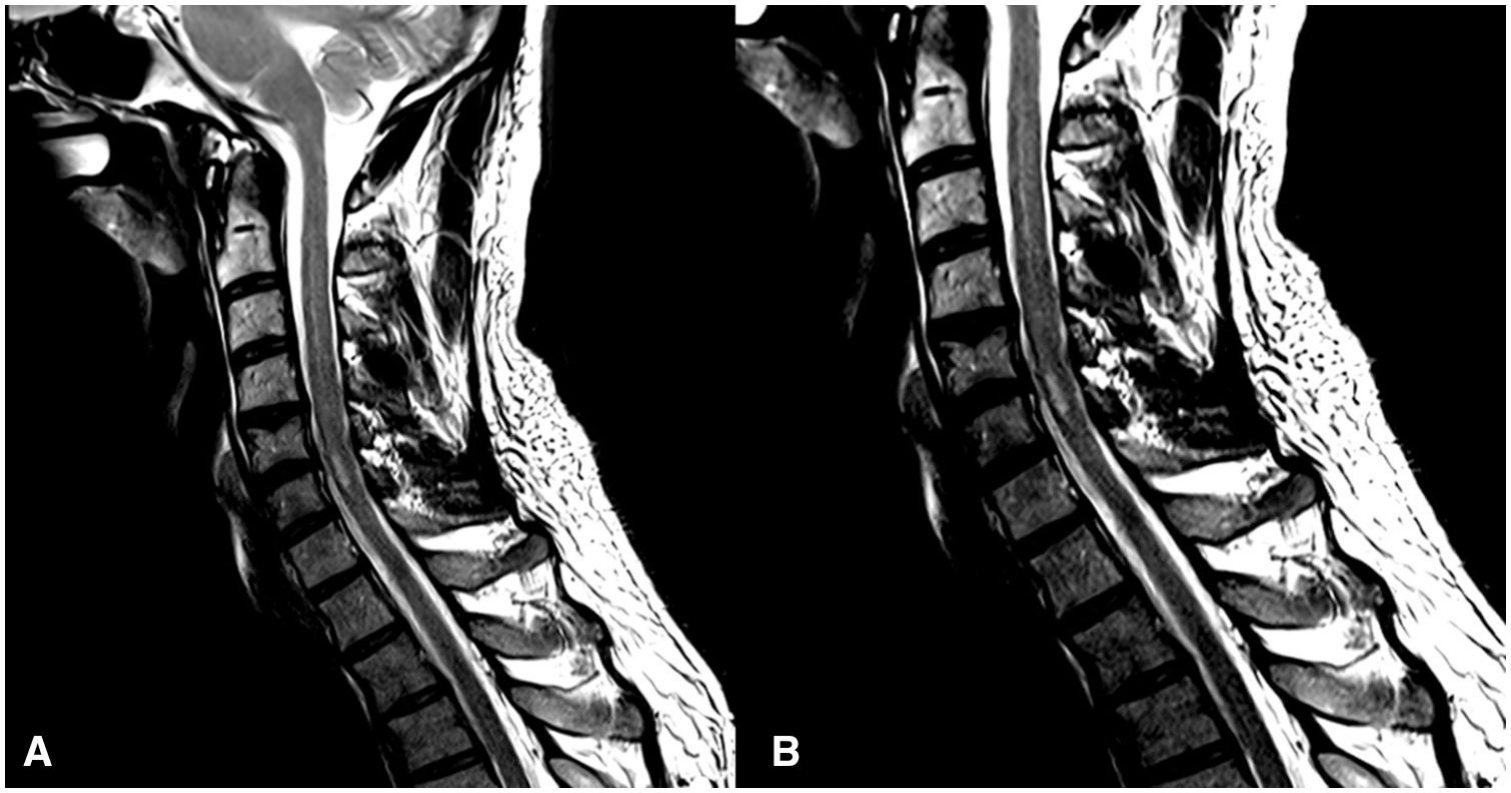
(A and B) Sagittal T2-weighted MRI cervical spine demonstrating FLAIR hyperintensity reflecting the C5-6 SCI.

**Figure 2.**
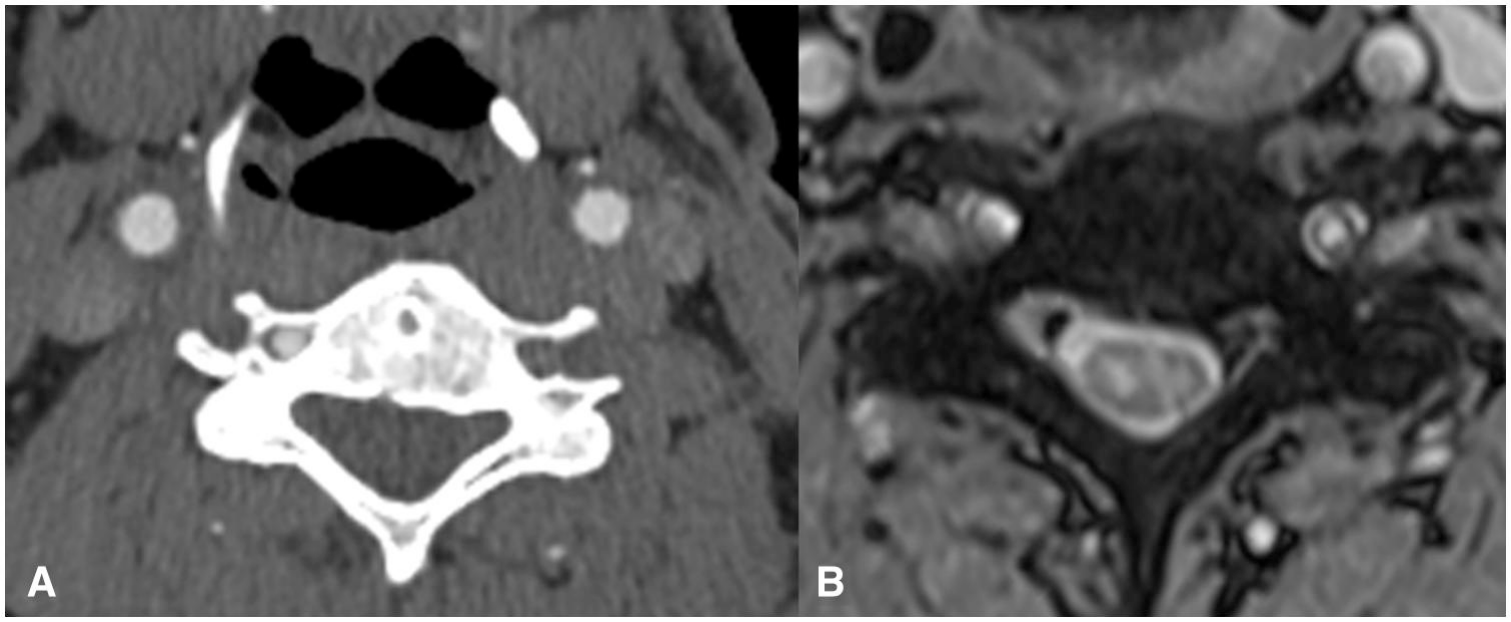
(A) CTA of the neck demonstrating loss of flow in the left vertebral artery. (B) Axial T2 MRI at C5-6 demonstrating right anterior SCI. The concurrent faint brightness, which is less pronounced in the left hemi-cord, is not represented on alternative views and was concluded to be artifactual at the multidisciplinary neuroradiology meeting.

**Figure 3.**
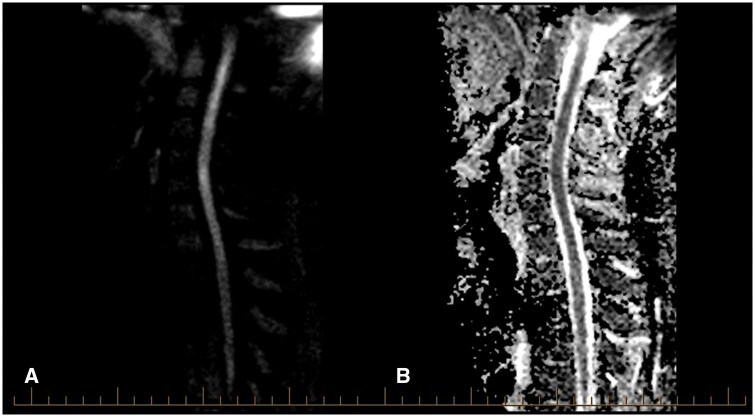
(A) Sagittal Diffusion Weighted MRI demonstrated a spinal cord infarct. (B) Matched sagittal MRI ADC demonstrating CSI.

**Figure 4.**
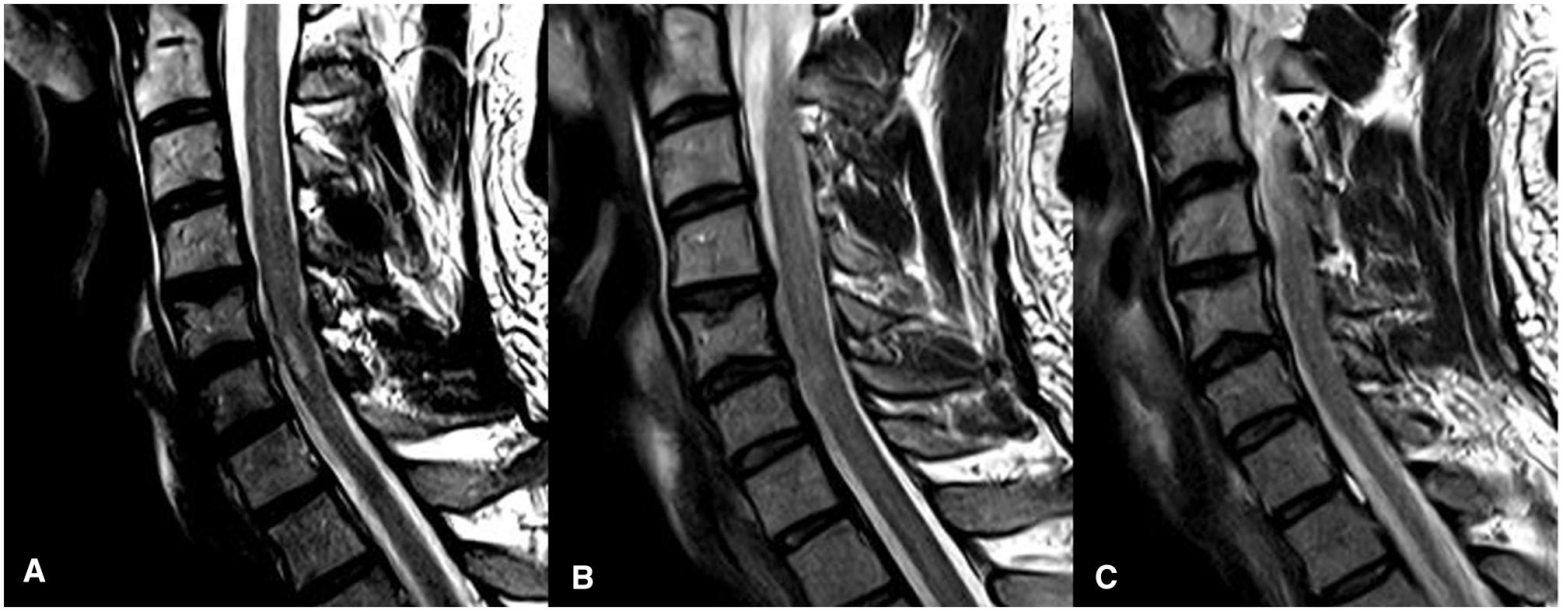
T2-weighted MRI of cervical spinal cord imaged at (A) right hemi-cord, (B) midline, and (C) left cervical hemi-cord demonstrating isolated FLAIR hyperintensity in the right cervical hemi-cord.

**Figure 5.**
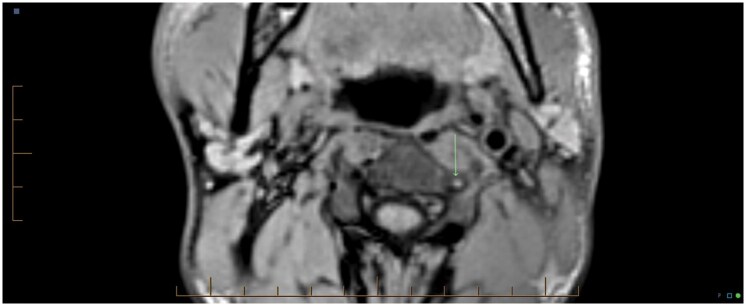
Axial T1 Fat Saturation image demonstrates loss of low signal and total occlusion of the left vertebral artery. No high signal crescent sign indicating intramural haematoma or arterial dissection.

His MR-Angiogram head and neck demonstrated left vertebral artery occlusion with loss of flow void from the origin at the left subclavian artery into the proximal intradural segment with reconstitution of flow in the distal intradural segment. There was no associated T1 hyperintense mural haematoma to suggestion a dissection (Figure 5). The MRI of the spine showed a focal abnormal T2 hyperintensity in the central grey matter of the cervical cord between C5 and C6 with a right-sided predominance, with mild diffusion restriction and postcontrast enhancement (Figures 1, 2B, 3A,B). The apparent left hemi-chord involvement was thought to be artifactual and was not present on sagittal section through the cord (Figure 4). The MRI brain did not demonstrate any demyelination, mass or stroke. Collating data from all his radiographic imaging, it was agreed that atheromatous vertebral artery occlusion was more likely than dissection.

During his admission, he intermittently had paraesthesia with decreased sensation down the medial aspect of the right upper limb. He also had one episode of recrudescence of severe pain after having a hot shower, which lasted one hour, settling with pregabalin and oxycodone. There was no recurrence after being advised not to bend his head and to avoid hot showers. It was considered that this may be Lhermitte’s and Uthoff’s phenomena.

**Figure 6.**
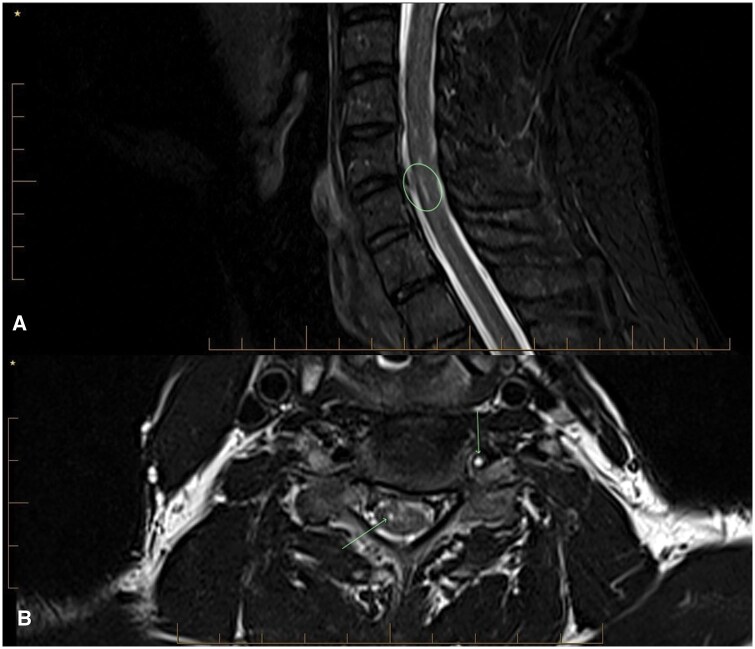
Follow-up imaging was completed 8 months post-index event. (A) MRI cervical spine Sagittal STIR image demonstrates linear T2 hyperintensity at C56 from prior right cord infarct. (B) MRI cervical spine Axial T2 image demonstrates T2 hyperintensity in the right central grey matter from a prior infarct. The left vertebral artery remains totally occluded with no flow signal.

The patient was discharged after 5 days on pregabalin, aspirin, and atorvastatin. At the time of clinic follow up his pain was almost entirely resolved with only very mild and occasional aching; he had experienced no further severe pain episodes, and no persistent neurological deficits related to his cervical spinal cord infarction. Follow-up 8 months post-event, the patient was similarly improved with nil recurrence and follow up imaging in Figure 6. demonstrates the radiological changes at that point.

## Discussion

### Spinal arterial blood supply

Blood supply to the spinal cord (SC) is complex and displays considerable anatomical variation. The major supply vessels are the anterior spinal artery, which runs the length of the cord in the anterior sulcus and 2 posterior spinal arteries. The anterior spinal artery (ASA) is initially formed from 2 branches of the vertebral artery at the V4 level and receives collateral supply from the radiculomedullary arteries at various levels. The ASA supplies the anterior two-thirds of the cord (corticospinal and spinothalamic tracts) and the paired posterior spinal arteries (PSA), the posterior one-third, which contains the posterior columns.^6^

Embryologically, at every spinal segment, there are bilateral metameric radicular arteries from the vertebral arteries, intercostal, and lumbar arteries, which supply the dorsal and ventral nerve roots as well as the spinal cord. Many of the radiculomedullary arteries (radicular arteries supplying the SC) regress, leaving several or a singular unilateral enlarged artery as the major source of blood supply, exemplified by the artery of Adamkiewicz and the artery of Lazorthes. In the cervical cord, the ASA supplies the spinal cord from C1-C3 spinal levels; between 1 and 3 radicular arteries supply it from C4-C5 and the C6-T1 cord is supplied by a branch or branches of the costovertebral trunk by the deep cervical, or the thyrocervical trunk by the ascending cervical arteries.^6^^,^^10^

The ASA can be single or duplicated and is best considered as not a continuous artery but rather a series of anastomotic segments. ASA gives off sulcal branches as it descends, alternating as either right or left sulcal arteries, supplying the SC. Each sulcal artery supplies 2 spinal segments. Cervical SCI is rare due to this robust collateralization. Consequently, transient ischaemic events are more common in the cervical cord than full infarction.^11^

### Cervical spinal cord infarction and vertebral artery dissection

ASA infarction is the most frequent with diplegia/quadriplegia and spinothalamic sensory loss.^11^ Bilateral hand weakness alone can occur or rarely an “man in the barrel syndrome”.^12^ Prognosis is generally favourable due to the cervical cord’s extensive collateral supply. A hemi cord infarction from sulcal artery occlusion is the next most common, with hand weakness or hemiplegia and contralateral spinothalamic loss, a subtype of Brown-Sequard Syndrome. Posterior artery infarction is the rarest, attributable to dual posterior spinal arteries, and manifests with unilateral or bilateral proprioceptive loss.^5^

Tan et al reported three patients with spinal sulcal syndrome (SSS) and retrospectively reviewed the literature of the 17 other reported cases. 94% of patients experienced acute pain at onset. Most had cervical cord infarction (90%), and 87% showed good functional recovery. VAD was the commonest cause (55%), with atherosclerosis and embolism accounting for the rest. The authors concluded that pain at the onset of sulcal artery syndrome with high cervical cord infarction (C2-4) was most likely due to vertebral artery dissection.^4^ To account for hemi-cord infarction, it is postulated that vertebral artery occlusion can: (1) result in embolism from VA thrombosis to sulcal arteries; (2) cause watershed ischemia to sulcal arteries as they are end arteries with no collaterals; (3) in the case of ASA duplication, result in unilateral radiculomedullary artery occlusion secondary to VAD.

In our patient with a lower cervical cord infarction of C5-6 as seen in Figures 1A,B, 2B, 3A,B, 4A the vertebral artery occlusion may have been due to either atherosclerosis or dissection. The mechanism of the SCI most probably involved an occlusion of the dominant unilateral radiculomedullary artery of the VA with a watershed effect or embolism to the sulcal artery. This pain-predominant syndrome is unique to the SCI literature; the only similar case is SSS with a rapidly reversible motor defect over 24 hours. We hypothesize that the forced right arm dominance due to the neonatal left Erb’s palsy may have led to increased synaptic connections in the right anterior spinal horn motor neurones, ameliorating the neurological effects of ischemia.^13^ This postulation regarding the aetiology of this atypical presentation raises the consideration for the role of further research into neuroplasticity, using those with neonatal spinal cord injury as a case-cohort, particularly as fields such as fMRI improve paired with EMG. A secondary postulated mechanism for this essentially pain-only presentation is the possibility of weakness and sensory change with an identifiable level at the index presentation, which had resolved over the 9 days before detailed neurological assessment occurred.

This case report highlights the importance of imaging the vertebral arteries and spinal cord in young patients with sudden onset cervical radicular pain with neurological deficits. SCI can mimic demyelination if an appropriate history and imaging sequences are not undertaken. This highlights the importance of including appropriate imaging sequences early, with the utility of DWI clear with its rapidity of acquisition and impact on diagnostic decision tree navigation, stressing its role in suspected acute myelopathies. Moreover, it highlights the significance of considering this differential in young patients presenting with cervical, upper limb and upper thoracic pain, and the importance of awareness of SCI pain syndromes.

## Learning Points

- Spinal Cord Anatomy: focus on cervical vasculature and pathological mechanisms for disruption.
- Spinal Cord Infarction syndromes.
- Neuro-remodelling may cause atypical presentations.

## Funding

None declared.
